# Prediction of 1p/19q Codeletion in Diffuse Glioma Patients Using Pre-operative Multiparametric Magnetic Resonance Imaging

**DOI:** 10.3389/fncom.2019.00052

**Published:** 2019-07-30

**Authors:** Donnie Kim, Nicholas Wang, Viswesh Ravikumar, D. R. Raghuram, Jinju Li, Ankit Patel, Richard E. Wendt, Ganesh Rao, Arvind Rao

**Affiliations:** ^1^Department of Bioinformatics and Computational Biology, University of Texas MD Anderson Cancer Center, Houston, TX, United States; ^2^Department of Computational Medicine and Bioinformatics, University of Michigan, Ann Arbor, MI, United States

**Keywords:** multiparametric MRI, image perturbation, radiomic features, glioma, persistent homology, 1p/19q codeletion

## Abstract

This study compared the predictive power and robustness of texture, topological, and convolutional neural network (CNN) based image features for measuring tumors in MRI. These features were used to predict 1p/19q codeletion in the MICCAI BRATS 2017 challenge dataset. Topological data analysis (TDA) based on persistent homology had predictive performance as good as or better than texture-based features and was also less susceptible to image-based perturbations. Features from a pre-trained convolutional neural network had similar predictive performances and robustness as TDA, but also performed better using an alternative classification algorithm, k-top scoring pairs. Feature robustness can be used as a filtering technique without greatly impacting model performance and can also be used to evaluate model stability.

## Background

1p/19q codeletion, is a genetic loss event that is somewhat rare in gliomas (Fuller and Perry, 2005; Eckel-Passow et al., 2015). It involves the complete deletion of the short arm of chromosome 1 alongside the deletion of the long arm of chromosome 19. Patients with this genetic loss event have been shown to have markedly improved prognosis and overall survival as compared to patients without 1p/19q codeletion (Boots-Sprenger et al., 2013; Cairncross et al., 2013; Van M den et al., 2013). The ability to identify patients from radiologic imaging would help to tailor treatment for this subtype of brain cancer.

Radiomics is the study of tumor imaging data, and the use of the imaging features to predict prognosis or genetic markers of these tumors. Radiological studies are standard of care for most cancer patients, but genetic profiling is available only for a subset of cancer patients (Gillies et al., 2015). Thus, understanding the relationship between tumor appearance on magnetic resonance imaging (MRI) and the genetic profile of a tumor could help to predict prognosis or to subtype tumors and thereby deliver more precise care to larger patient populations.

A number of publicly available datasets and toolkits exist for measuring texture-based features on tumors (Clark et al., 2013; van Griethuysen et al., 2017). However, while there has been progress in measuring these features, there is some concern about the robustness and generalizability of radiomic features. Other studies on CT scans have shown that some texture-based features are not stable under perturbation in test-retest comparisons (Bogowicz et al., 2016; van Timmeren et al., 2016). In order further to assess the degree of instability, this study has investigated the effect of image perturbations on additional feature types beyond texture, and their eventual effect on classification power in MRI scans.

## Methods

A set of brain MRI data were drawn from the MICCAI BRATS 2017 challenge dataset (Menze et al., 2015; Bakas et al., 2017a, 2018). The multimodal Brain Tumor Image Segmentation Benchmark (BRATS) 2017 dataset was originally designed for the brain tumor segmentation challenge and comprises pathologically confirmed LGG (*n* = 65) and HGG (*n* = 102) cases from The Cancer Imaging Archive (TCIA) (Bakas et al., 2017b,c). The dataset contains pre-operative multimodal MRI sequences, namely T1, T1-post, T2, and FLAIR, and was acquired with differing imaging/clinical protocols and scanners from 19 different institutions. All tumor volumes in the imaging dataset had been segmented manually by one to four different experienced neuroradiologists.

Genetic markers for this TCIA dataset were gathered from The Cancer Genomics Archive (TCGA). The patients were first retrospectively identified with histologically confirmed WHO grade II-IV gliomas (*n* = 1,122) and their corresponding 1p/19q chromosome codeletion statuses (after surgical biopsy). In addition, the patients' age, gender, Karnofsky Performance Score (KPS) were collected as clinical variables.

These four sequences were co-registered to the T1 post-sequence as it had the highest spatial resolution. They were then resampled to 1 × 1 × 1 mm isotropically in an axial orientation by using a linear interpolation algorithm. Then, all images were skull-stripped to anonymize the patient information and remove extraneous regions of the scan (Bauer et al., 2012).

The scans were prepared by performing N4 bias correction, normalizing intensity values by interquartile range, and cropping and reshaping to the volume of interest. Normalization of the intensity was performed based on the interquartile range for a particular modality of the non-tumor brain volume. The slices were resampled to a 142 × 142 image size that was cropped to the tumor area of interest. This methodology is similar to that used by Chang et al. (2018a) in order to provide the type of input that the neural network anticipated.

The breakdown of the dataset for 1p/19q codeletion vs. non-codeleted cases was heavily skewed toward the non-codeleted cases, with 13 cases with codeletion and 130 without codeletion. As such, the codeleted cases were heavily oversampled in slice selection at a 20:3 ratio to achieve a closer balance of class ratio. The largest 20 image patch slices for each codeleted scan was taken. For the non-codeleted scans the 50, 75, and 100th percentile slices (based on size) were taken.

The dataset was split patient-by-patient into sets of 80% for training and 20% for testing. This preserved the class ratio in the training and testing sets, as the number of positive cases was so low. This process was repeated 10 times independently for a total of 10 independent splits. Each of these independent splits had the entire analytic process performed to assess the robustness of the results. The training set was used in 5-fold cross-validation for each of the models, where patients were kept together in the cross-validation folds.

**Figure 1:**
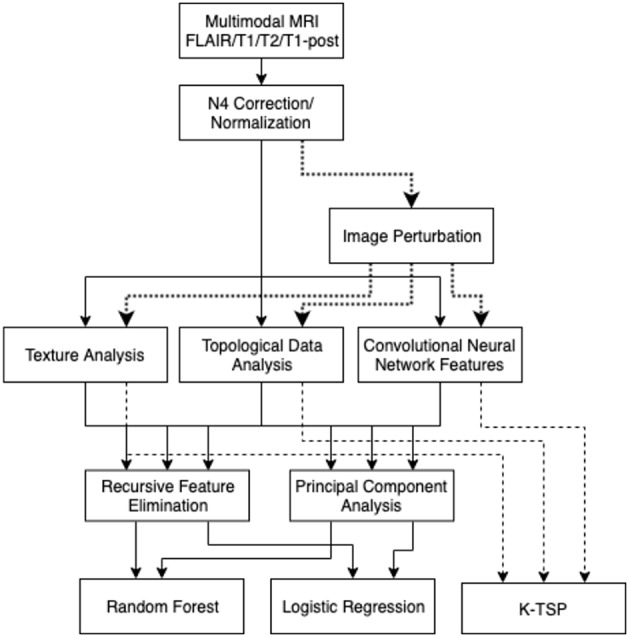
Analysis pipeline: images are normalized, then the three types of features were collected. These features are filtered with RFE and PCA, then used to build a random forest model or logistic regression model. Image perturbations are used as an additional filter by including only relatively robust features. The kTSP algorithm used the same feature set to build its predictions.

The three types of features measured in these scans were texture-based features, persistent homology topological features, and features based on a pre-trained convolutional neural network (Figure 1). The texture features were extracted slice-by-slice using the Pyradiomics package (van Griethuysen et al., 2017). The types of features were based on the tumor region of interest on each of the modalities. The texture features that were extracted included: first-order intensity features, shape features, gray-level co-occurrence matrix features (GLCM), gray-level run length matrix features (GLRLM), gray level size zone matrix features (GLSZM), and neighboring gray-tone difference matrix features (NGTDM).

**Figure 2:**
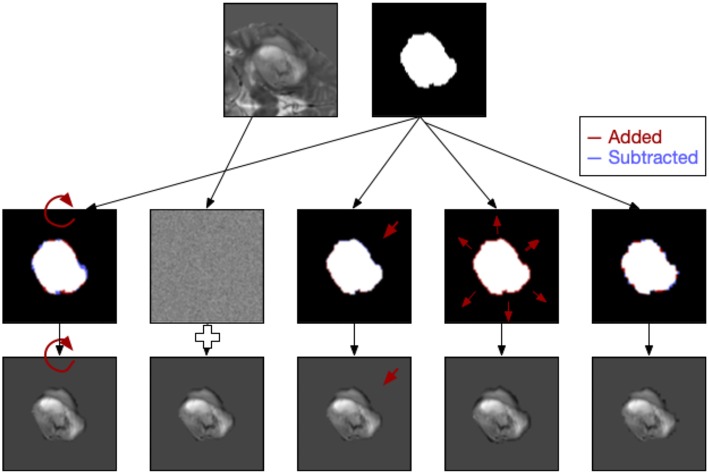
Examples of five types of image perturbation on a slice of the tumor (rotation, noise addition, translation, volume alteration, and contour alteration).

It is well-known that MRI studies suffer from a variety of noise sources, so the underlying integrity of the image data carries some uncertainty. A topological approach was evaluated to see if the features generated were less susceptible to this uncertainty than traditional texture-based approaches. These topological features were based on persistent homology and how the topology changes with shifts in the image intensity threshold. Barcodes describe when a connected component or tunnel was created and destroyed by this shifting threshold (Figure 2; Adcock et al., 2014). These barcodes were collected with the GUDHI python package (Maria, 2015). These barcodes were characterized by their polynomial features, along with statistical features about their birth and death intensities, bar lengths, and death intensity distribution. These features were based on work in Adcock et al. (2013) and Giansiracusa et al. (2017).

A pre-trained convolutional neural network (CNN) was used to calculate deep learning-based features, from Chang's work on IDH1 mutation (Chang et al., 2018a). Chang's model was useful for this investigation as it focused on gliomas and featured the same MRI modalities as were present in this study (T1, T2, FLAIR, and T1-post). The second to last layer of the network was used to extract features rather than to feed into a softmax layer to predict IDH1 mutation. We expected the network to produce some features that are relevant to this 1p/19q dataset because the current work was of the same fundamental nature as the problem in Chang's work.

Two versions of feature reduction/selection were evaluated in the training set of this study: recursive feature elimination (RFE) and principal component analysis (PCA). RFE was performed with 10-fold cross-validation, to determine the optimal number of features (k), then the k best features were selected. PCA was performed and a cutoff of 95% cumulative variance was used to cull the insignificant components in PCA reduction.

Each of the feature sets—texture, topology, and CNN—had feature selection performed, and then those features were fed into a random forest model and a logistic regression model. The models were tuned using 5-fold cross-validation with folds that kept patients within the same fold. The random forest models were optimized over a number of hyperparameters including: tree counts of 200–2000, maximum depths of 10–100, the minimum sample split, and minimum leaf size. The logistic regression models had normalization hyperparameters of L1 vs. L2 normalization, and regularization strength from 10^−3^ to 10^5^.

The models were evaluated primarily on the held-out 20% testing set, where area under the receiver operator curve (AUROC), accuracy, sensitivity, and specificity were measured. Additionally, combined models, which used features from texture, topology, and CNN, were also tested using the same approach. The clinical patient characteristics (age, sex, and Karnofsky performance score) were tested independently to gauge their performance in comparison to the imaging-based features.

### Robustness of Features

Each of the image slices was perturbed using image processing techniques to produce relatively small changes to the image following the approach of Zwanenburg et al. (2019). Five classes of perturbation were performed on the images: image rotation (R), image translation (T), image Gaussian white noise addition (N), mask volume alteration (V), and contour randomization (C). Images and masks were rotated around the mask center of mass to approximate changes in head position in the scanner. Image translations involved subpixel shifts which resampled the images on new slightly modified coordinate systems. Image noise addition added randomized Gaussian noise based on the noise levels of the original slice. Volume alteration grew or shrank the mask based on the Euclidean distance transform and the percentage of volume added or subtracted. Lastly, contour randomization combined superpixel segmentation of the underlying image with a probabilistic selection of those superpixels based on their overlap with the mask to produce altered contours (Figure 2).

Each of the altered images then had its texture and topological features evaluated for the range of individual perturbations. For each category of perturbation and each feature, the intraclass correlation coefficient (ICC) was calculated to determine the variability or robustness of that feature to the perturbation in question. After calculating the ICC, any feature that had an ICC of <0.75 for any of the perturbations was excluded from this round of modeling. With that filter in place, the same modeling procedure was followed to evaluate the predictive power of texture and topological features across the 10 instances.

### Classification With K-top Scoring Pairs

As an additional analysis, the same texture, topological, and CNN features were used to train a model using the k-top scoring pairs algorithm (kTSP). The kTSP algorithm classifies samples by identifying k-pairs of features whose relative expressions/values are inverted between the categories, i.e., it tries to find pairs of genes A and B whose relative rankings are inverted in most samples of the two cases. This gives an easy to interpret decision rule and makes the classifier robust to data normalization procedures. Given that different measurement technologies have different dynamic ranges, classifiers based on relative rankings of features rather than their absolute values are highly valuable for integrating and comparing across multiple sources of data.

The extracted CNN, textural, and topological features were used to train a kTSP classifier for predicting patient 1p/19q codeletion status using the switchbox R package (Afsari et al., 2015). Since kTSP is a greedy algorithm, we retained only features that were measured to be significantly differential between the two classes (Wilcox test *p* < 0.1 after BH correction). We then split the data into training and test sets (70:30 split) and estimated classifier performance by measuring training and test set roc values. Since the codeletion cases were heavily resampled, we grouped features from the same patient together while doing the train/test split so as to ensure that training and testing cases are really independent. By repeating this procedure for a total of 5,000 times and building classifiers with k allowed to range between 3 and 15 pairs of features, we estimated the 95% highest posterior density intervals for train and test AUC values for classifiers built from the three datasets.

## Results

**Figure 3:**
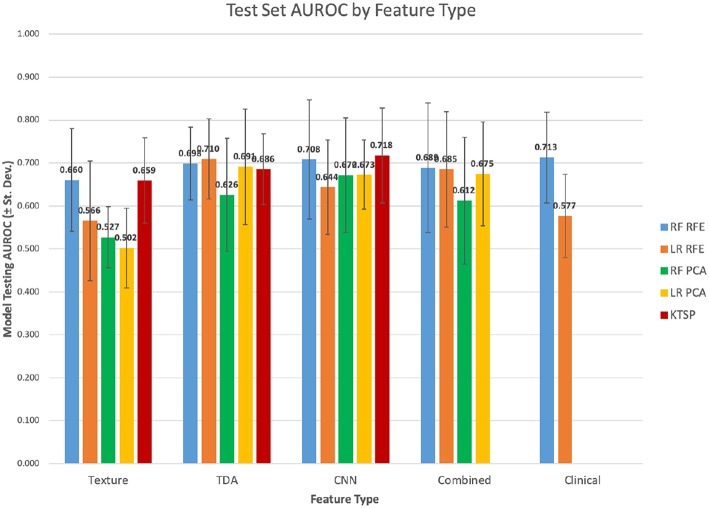
Test set mean AUROC by feature type.

Texture features were evaluated across the 10 independent train/test splits to measure their predictive power (Table 1; Figure 3). PCA-based feature reduction on texture features did very poorly on the test set with an average AUROC across the 10 train/test splits of 0.502 with linear regression (LR) and 0.527 for random forest (RF). RFE achieved test set AUROC values of 0.660 and 0.566 for LR and the RF models, respectively. However, the standard deviation of AUROC across the different splits was quite high (0.120, 0.139), suggesting that with a small dataset, the models' performance can be somewhat unstable.

**Table 1:** Test set statistics across 10 independent splits.

	**AUROC**	**STD of AUROC**	**Sensitivity**	**Specificity**	**Accuracy**
Texture only RF RFE	0.660	0.120	0.782	0.558	0.669
Texture only LR RFE	0.566	0.139	0.775	0.479	0.629
Texture only RF PCA	0.527	0.071	0.543	0.644	0.581
Texture only LR PCA	0.502	0.093	0.573	0.610	0.583
TDA only RF RFE	0.698	0.085	0.653	0.738	0.682
TDA only LR RFE	0.710	0.094	0.723	0.675	0.692
TDA only RF PCA	0.626	0.132	0.647	0.648	0.638
TDA only LR PCA	0.691	0.135	0.677	0.694	0.676
CNN only RF RFE	0.708	0.139	0.905	0.546	0.727
CNN only LR RFE	0.644	0.110	0.775	0.565	0.669
CNN only RF PCA	0.672	0.133	0.627	0.750	0.675
CNN only LR PCA	0.673	0.081	0.823	0.546	0.686
Combined RF RFE	0.689	0.150	0.877	0.552	0.714
Combined LR RFE	0.685	0.135	0.770	0.638	0.700
Combined RF PCA	0.612	0.148	0.655	0.637	0.638
Combined LR PCA	0.675	0.121	0.865	0.525	0.698
Clinical per patient RF	0.713	0.106	0.667	0.854	0.800
Clinical per patient LR	0.577	0.097	0.467	0.819	0.759

*Darker blue indicates improved AUROC*.

Features from topological data analysis were also evaluated across the 10-independent training/testing splits (Table 1). In this case, most of the analyses performed relatively similarly in terms of AUROC, ranging from 0.626 to 0.710 for these different models with topological features. Again, the standard deviation of AUROC across the different training/testing splits was relatively broad (0.085–0.135), though slightly lower than that of the texture features. Texture and TDA features overall had relatively similar performance, with a slight edge to TDA features, though well-within the variability of these statistics.

When modeled using random forests or logistic regression, the CNN feature set had similar predictive performance to topological features (Table 1). The AUROCs of these models fell between 0.644 and 0.708. It also performed similarly with the k-top scoring pairs (kTSP, Table 2) approach when compared to the random forest (RF) or logistic regression (LR) with an average AUROC of 0.718. Combining the three feature types neither improved or decreased performance, suggesting that they were not measuring vastly different types of information.

**Table 2:** Test set statistics for kTSP algorithm.

	**AUROC**	**STD of AUROC**
Texture only kTSP	0.659	0.099
TDA only kTSP	0.686	0.083
CNN only kTSP	0.718	0.111

*Darker blue indicates improved AUROC*.

Overall, RFE somewhat outperformed PCA as a feature selection tool, although the scale of the difference depended on the feature set. Logistic regression had similar results to random forest classification in most cases, although there were some exceptions.

**Figure 4:**
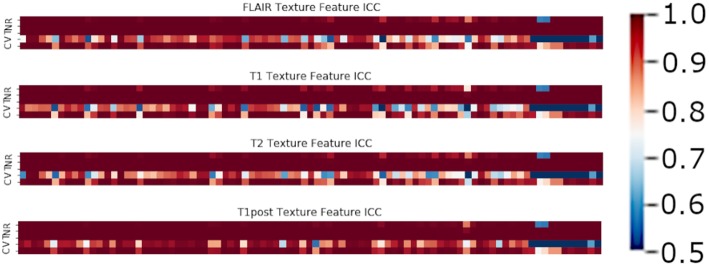
Mean ICC of Texture features (R, Rotation; N, Noise addition; T, Translation; V, Volume alteration; C, Contour alteration). Volume based perturbations had the largest effect on the robustness of texture features, followed by contour alteration. There was a range of ICC values for the different features.

**Figure 5:**
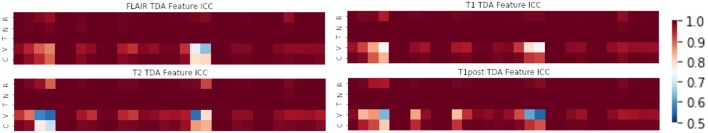
Mean ICC of TDA features (R, Rotation; N, Noise addition; T, Translation; V, Volume alteration; C, Contour alteration). Volume based perturbations had the largest effect on ICC for topological features. Polynomial features 3 and 4 were the least robust to perturbation, while other TDA features were relatively stable.

**Figure 6:**
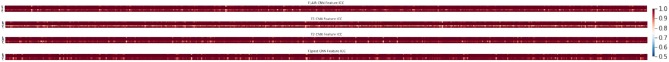
Mean ICC of CNN features (R, Rotation; N, Noise addition; T, Translation; V, Volume alteration; C, Contour alteration). CNN based features were broadly stable to perturbations, though still most affected by volume changes.

In terms of feature robustness, topological features had much better ICC after perturbation than did the texture-based features. Of the 356 texture features, an average of ~117 features (32.8%) had an ICC of <0.75 on the perturbations and were excluded from this round of modeling (Figure 4). Of the 120 topological features, an average of ~10 (8.1%) had an ICC of <0.75, as such most of the features were included in the next round of modeling (Figure 5). Only an average of ~3 of the CNN features (0.15%) were excluded at the 0.75 ICC cutoff (Figure 6).

**Figure 7:**
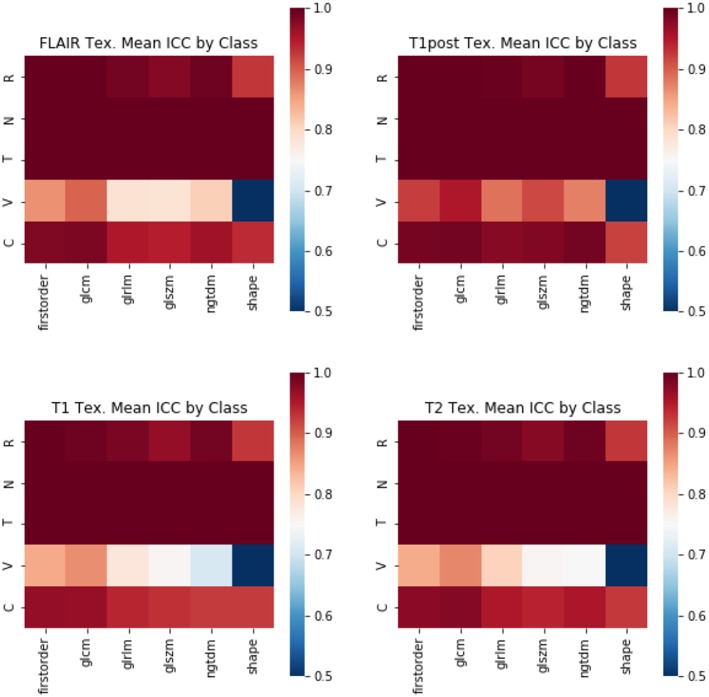
Mean ICC of Texture features by feature class. (R, Rotation; N, Noise addition; T, Translation; V, Volume alteration; C, Contour alteration). Changes in volume had the largest effect on the stability of radiomic features. The least stable class of features were the shape-based features, whereas GLCM and first-order features were more stable.

The perturbation types which had the lowest average ICC were volume perturbation and contour alteration. Noise addition and translation had little impact on the ICC values for texture and TDA features. Volume alteration, and contour alteration both affect the segmentation mask of the tumor without an impact on the underlying image. This does, however, affect the region investigated by topological and texture features. Notably, when looking at stability in texture features by the class of feature, shape-based features performed poorly under volume-based alterations and were affected by rotation more than the other classes (Figure 4). Overall, GLCM-based measures were the most stable of the texture features as a class under these perturbations (Figure 7). Among the TDA features, polynomial features 3 and 4 were the least robust to perturbation, suggesting higher order polynomial features are less stable than lower order features (Figure 5).

**Figure 8:**
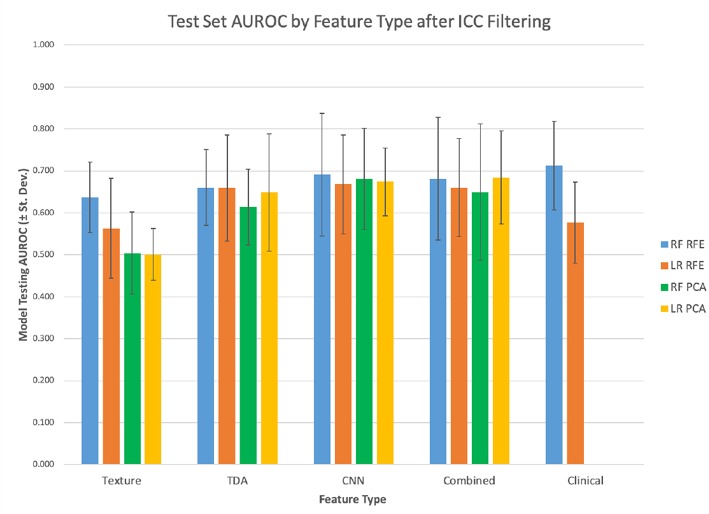
Filtered features test set mean AUROC.

Features that had a low ICC were excluded and the models were retrained on the reduced feature set. Then the predictive power of these models was measured on the testing set. Overall, when excluding non-robust features from modeling, the performance of the models dropped slightly in terms of AUROC, although most had relatively similar power (Table 3; Figure 8).

**Table 3:** Test set statistics, after exclusion of unstable features.

	**AUROC**	**STD of AUROC**	**Sensitivity**	**Specificity**	**Accuracy**
Texture only RF RFE	0.637	0.084	0.758	0.569	0.661
Texture only LR RFE	0.563	0.120	0.775	0.512	0.644
Texture only RF PCA	0.505	0.097	0.552	0.625	0.577
Texture only LR PCA	0.501	0.062	0.532	0.625	0.568
TDA only RF RFE	0.660	0.090	0.685	0.635	0.652
TDA only LR RFE	0.659	0.126	0.635	0.712	0.662
TDA only RF PCA	0.614	0.090	0.767	0.569	0.649
TDA only LR PCA	0.649	0.140	0.655	0.613	0.627
CNN only RF RFE	0.691	0.146	0.870	0.567	0.721
CNN only LR RFE	0.668	0.118	0.867	0.510	0.692
CNN only RF PCA	0.681	0.121	0.725	0.644	0.679
CNN only LR PCA	0.674	0.081	0.847	0.531	0.691
Combined RF RFE	0.681	0.146	0.860	0.552	0.707
Combined LR RFE	0.660	0.117	0.830	0.540	0.687
Combined RF PCA	0.650	0.163	0.760	0.619	0.686
Combined LR PCA	0.684	0.111	0.835	0.569	0.703

*Darker blue indicates improved AUROC*.

**Figure 9:**
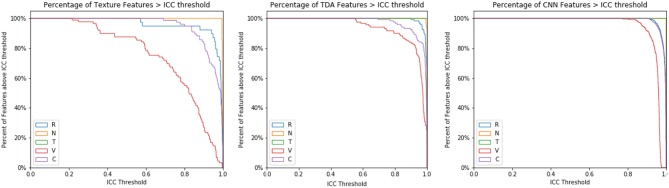
ICC Threshold effect by perturbation type. The ICC cutoff for features is varied from 0 to 1, and the percentage of features that survive each threshold is recorded by perturbation type.

Increasing the ICC cutoff would increase the number of features excluded from the analysis. Thus, this effect was further studied for each type of image perturbation (Figure 9). Texture features are broadly susceptible to contour and volume alterations. A subset of texture features was susceptible to rotation effects as well, although very few features were affected by the noise or translation perturbations. CNN features had a relatively narrow range of ICC values, and TDA features were broadly stable, though a subset of TDA features were less robust.

## Discussion

In this study, topological data features performed as well as or better than texture features in predicting 1p/19q codeletion status. However, model performance varied across the different training and testing splits of the data, as evidenced by the standard deviation of model performance. CNN-based features also had similar performance to topological features with random forest and logistic regression, but they performed notably better with kTSP as the modeling algorithm.

One concern, however, is the relatively small sample size of 143 patients, of whom only 13 had the 1p/19q codeletion. This may be a large factor in the uncertainty in the prediction estimates. Oversampling the 1p/19q codeletion alleviates the class imbalance somewhat, but raises some concerns about overfitting, especially in models like random forest. Finding additional MRI studies with confirmed 1p/19q codeletion would improve the generalizability of any models derived from this data.

The kTSP algorithm is more often used in gene expression array data but can be applied just as easily to other large-scale datasets. By finding pairs of features that have different relative orderings in the two sets, kTSP is less dependent on the absolute magnitude of change than are the other methods. It also benefits from having a large number of features to search that have positive and negative associations with the target classification. As the CNN features are not human-designed features, and there is a larger set of CNN features with more variability in direction, kTSP seems to take better advantage of these features than features like TDA or texture.

Traditional radiomics features based on gray levels, such as GLCMs can be dependent on the number and boundaries of gray level bins. Volume and contour-based alterations affect the set of pixels under investigation, which could heavily influence the resulting texture matrices. Topological barcodes have been found to be mostly stable under image-based perturbations of the data, as have the CNN-based features from this pre-trained model.

While other groups have also used radiomic features or neural networks to predict 1p/19q codeletion, this paper seeks to compare multiple potential approaches (Han et al., 2018; Lu et al., 2018; Zhou et al., 2019). Other papers have trained neural networks to predict 1p/19q codeletion, whereas this study only used a pre-trained neural network on the dataset (Akkus et al., 2017; Chang et al., 2018b). One weakness of this approach was that the testing AUROCs of the models in this study were not as high as some that have been reported in other studies. However, this study was also able to evaluate the robustness of these features through image perturbation. Additionally, the models in this study incorporated topological features based on persistent homology, which had better performance than radiomic features and were more stable to perturbation.

Clinical value is more difficult to assess than statistical significance, as it is dependent on the prognostic value of the biomarker, the current standard of care, and the predictive power of the model. 1p/19q codeletion is typically evaluated through genetic testing of a tissue sample, whereas the benefit of a radiogenomic approach is to evaluate the imaging markers of a tumor without biopsy or resection. However, as many glioma patients receive a biopsy for diagnostic purposes, a radiogenomic model would have to be exceptionally predictive to warrant replacement of this procedure. This study aims more to understand the types of features radiogenomic approaches are detecting, and how robust they are in different conditions rather than to replace the test.

## Future Directions

While this study used the image perturbation parameter space of the Zwanenberg paper, it would be worthwhile to tune the tested space of parameters further. The level of noise is based on wavelet estimation, but by visual inspection is not apparent until the noise level is increased by 1–2 orders of magnitude. Additional levels of noise could be investigated, as could the types of noise, such as changing the noise to a Rician distribution or adding the noise to k-space rather than the image domain. However, as these perturbations take each measurement and multiply it out by orders of magnitude, the computational demands can add up quickly. Thus, there is a tradeoff between perturbation complexity, the size of the parameter space, and the certainty of the resulting robustness measure.

Further investigation of the robustness of these measures could be done by simulating scans from the underlying physics, using a Bloch equation simulator (Ford et al., 2018). This would allow for measuring the effect of variable image collection parameters such as TE, TR, and field strength. Understanding these effects would help to account for concerns about variability in the underlying MRI protocols. Unfortunately, these simulations are primarily of normal brain images, so may not fully reflect the interaction between tumor tissue alteration and image feature robustness.

## Data Availability

Publicly available datasets were analyzed in this study. This data can be found here: https://www.med.upenn.edu/sbia/brats2017/data.html.

## Ethics Statement

Human subjects data in the form of medical imaging data, and genetic information was collected from The Cancer Imaging Archive (TCIA) and The Cancer Genome Atlas (TCGA). All data in these National Cancer Institute (NCI) databases are anonymized and, therefore, individual institutional IRB approval is not required for this retrospective review, although it should be noted that all data were originally submitted to TCGA and TCIA by the contributing institutions under an IRB-approved protocol. TCGA LGG tumors were included in this study if they had a corresponding diagnostic MRI study in TCIA.

## Author Contributions

DK: initial development of modeling pipeline, data analysis and data normalization, and editing of manuscript. NW: reworked pipeline, data analysis, added image perturbation, and drafted manuscript. VR: development of kTSP section of code/data analysis, writing, and editing. DR: data analysis and manuscript editing. JL: kTSP data analysis and manuscript editing. AP: design of study and manuscript editing. RW: design of study and major revisions to manuscript. GR: design and conception of study. AR: design and oversight of study, provided direction for modeling, image perturbation and kTSP analysis, and manuscript editing.

### Conflict of Interest Statement

The authors declare that the research was conducted in the absence of any commercial or financial relationships that could be construed as a potential conflict of interest.
